# Report of the Special Committee on Diseases of the Eye

**Published:** 1865-06

**Authors:** E. L. Holmes

**Affiliations:** Chicago


### CHICAGO MEDICAL EXAMINER.

N. S. DAVIS, M.D., Editor.

VOL. VI. JUNE, 1865. NO. 6.

### Original Contributions.

### ARTICLE XVIII.

### REPORT OF THE SPECIAL COMMITTEE ON DISEASES OF THE EYE.

By E. L. HOLMES, M.D., of Chicago.

Presented to the Illinois State Medical Society, May, 1865.

As Chairman of your Committee on Diseases of the Eye, I would most respectfully report that I have received no communications either from members of the profession at large, or of this Society. This is to be regretted, for diseases of the eye are so prevalent in this State, they are the cause of so much misery, that they are certainly of sufficient importance to awaken special interest in every one devoted to the relief of physical suffering. This subject should not attract the attention of specialists alone. Whatever may be said in reference to the necessity of specialties, it is true there are certain classes of diseases of the eye which should be carefully investigated by general practitioners in the country, for upon them alone can patients suffering from these diseases depend for aid. These classes of disease are important, because they are common, and, if neglected, so often result in blindness or injury of vision. Other classes of disease, on the other hand, require more experience in observation, and practice in their surgical treatment, than most medical men can possibly acquire in their ordinary routine of labors. The operation for extraction of cataract, for artificial pupil, the diagnosis of certain forms of diseases of the choroid or retina, and the use of the ophthalmoscope, require long and careful practice on the part of the student.

The science of medicine is too broad for any one to cultivate with success the whole of its wide domain. For this reason, no one can become really distinguished for skill and knowledge in all the different departments of medicine and surgery. On the contrary, nearly all those who have made most important discoveries, who have written works which are regarded as standard authorities in any branch of our science, have been those who have concentrated their energies upon one class of subjects. In Europe the position of specialists in our profession has been established beyond reach of criticism. In this country there seems to be less inclination on the part of the profession to recognize the necessity, or even advantage, of subdivision of labor in the practice of medicine. This prejudice exists, undoubtedly, in consequence of the ignorance and questionable character of a very large proportion of men engaged in the treatment of diseases of special organs. But specialties are no more responsible for this evil, than the science of medicine is responsible for the number of charlatans that pretend to worship at its altar.

The eye and ear are confessedly organs, of which the anatomy and physiology, with the treatment of their diseases, are not well understood by the vast majority of our profession. This arises, not from any particular difficulty of the subjects, but from a want of interest. The fact that a physician may become distinguished as a practitioner, and as a man of science, without a knowledge of ophthalmology, seems to foster this neglect of the latter, for it is well known that while affections of nearly every other organ affects the general health, comparatively few diseases of the eye or ear have much influence upon general diseases of the system. It seems scarcely just that members of the profession, who absolutely neglect the study of diseases of the eye and ear, should discountenance the efforts of those who find interest in ophthalmic science alone, sufficient to employ their best energies. Fortunately, as the profession recognizes the unlimited field of labor which the science of ophthalmology presents, there is less prejudice against well-educated specialists.

In reference to sympathetic ophthalmitis, and conjunctivitis, as it exists in this State, we would now add nothing to our former report, although we had hoped members of the Society would contribute some new facts regarding these diseases. At some future time, we may recall the attention of the Society to these subjects.

There are a few points in the diagnosis and treatment of iritis, which seem of sufficient importance to present to this Society. Inflammation of the iris is not an uncommon disease, and is not unfrequently misunderstood, and consequently maltreated by well-educated physicians. From the fact, that when left to nature, or when subjected to improper treatment, it almost invariably terminates in total or partial loss of vision, it is highly important that an early and correct diagnosis should be established. Those who have not had considerable experience in this disease, occasionally find difficulty in forming a diagnosis, from the fact that the disease is sometimes accompanied, almost from the first, with severe inflammation and œdema of the conjunctiva. One of the most important symptoms which can aid the practitioner in recognizing the disease, in many obscure cases, is the great indistinctness of vision, which cannot usually be accounted for by the degree of congestion of the mucous and sub-mucous membrane of the globe. When with this symptom, the character of the neuralgic pain in and around the eye, with the general appearance of the pupil, leave any doubt regarding the nature of the disease, a few drops of a solution of atropine, or one-eightieth of a grain of the salt itself, introduced between the lids, will usually remove all doubt, by the slow and irregular dilatation of the pupil.

The Society is well aware that, in the treatment of iritis, mercury has long been considered as the all-important agent. The experience, however, of several well-known oculists has shown that the disease may be treated successfully without it.

Although we have not discarded the remedy in the treatment of iritis, we are confident it is of secondary importance, as compared with atropia. This remedy should be applied either in the form of solution, or in substance, at the very earliest stage of the disease. It not only dilates the pupil, and thus prevents exudation from the pupillary edges of the iris from being deposited on the central portion of the lens, but it seems to exert a direct influence in reducing inflammation. Pain should be subdued, especially at night, by anodynes, either internally or in the form of sub-cutaneous injection near the eye. Although, instead of mercury, iodide of potash and turpentine have been used with great success by some, oculists generally have not ceased to place great confidence in mercury. The great objection to this remedy is not so much in its moderate use, as in its abuse. We are convinced, that with the early and free use of atropine, the profession will be inclined to administer mercury in much less quantities than usually given.

As the subject of cerebro-spinal meningitis has excited, of late, the attention of the profession, it may be a matter of interest to state that I have observed, during the past year, two cases of destructive choroiditis, dependent upon this disease. It is well known that several diseases, as small-pox, typhoid and puerperal fever, erysipelas, and serious affections of the heart, lungs, and liver occasionally develop acute or sub-acute choroiditis. We have already observed two cases of choroiditis, complicating severe erysipelas of the head and face.

In one of the former cases, in a child three years of age, the disease seemed to have been developed in one eye during the cerebral affection, and continued after the recovery from the primary disease as a sub-acute inflammation of the choroid and iris, which induced sympathetic ophthalmitis in the other eye. The child, when brought to my observation was totally blind.

In the other case, a boy seven years old was affected with choroiditis in the right eye. Six months after recovery from the primary disease, I found the globe slightly atrophied, and tender on pressure, with a calcareous lens attached to the iris. There was considerable irritation and photophobia in the other eye. Although, I believe, in a majority of such cases, the extirpation of the eye first affected is, in nearly every respect, preferable to any other operation, I removed a portion of the iris and extracted the calcareous mass through a lineal incision in the cornea.

Two cases of paralysis of the muscles of the eye, following diphtheritic affection of the throat, have fallen under my observation. In one case, a female of middle age had suffered a dangerous attack of diphtheria. During recovery, the muscles of the soft palate became paralyzed, and soon after the muscles of both eyes. Two months after the attack, the muscles of the throat had nearly regained their normal power. The muscles of the eyes, however, remained paralyzed; the globes were absolutely motionless; the upper lids retained their normal movements; the pupils were somewhat dilated, and the power of accommodation quite imperfect. Under the use of iodide of potash for three weeks, the affection of the eyes and throat disappeared.

In the second case, the paralysis was confined to the third pair of nerves, and branches of the seventh pair, of the right eye, causing ptosis with extreme external strabismus, and paralysis of the muscles of the right side of the face.

There are some obscure points in the connection between Bright's disease of the kidneys, and certain affections of the retina, which, we believe, merit the attention of the Society, as giving scope for investigation.

What form of albuminaria is most liable to complication with fatty degeneration of the retina? How large a proportion of patients affected with albuminuria suffer from amaurotic symptoms? It is worthy of notice that in these cases of retinitis, the affection of the kidney can be diagnosticated with almost absolute certainty, by the ophthalmoscopic appearances of the retina alone. During the past year, I have been consulted in two cases of amaurotic disease of the eye, in both of which the ophthalmoscopic appearance of the retina led to the suspicion that the patients were suffering from albuminuria. The examination of the urine confirmed the opinion.

There are several modifications in important operations upon the eye worthy of the attention of the Society, but I would prefer to report upon them at some future time. I will, however, briefly allude to a modification in the operation for the extraction of hard cataract. It is well known that two of the principal dangers in the extraction of hard cataract, consist in injury of the iris by the passage of the hard lens through the contracted pupil, and in the presence of fragments of cortical substance of the lens remaining in contact with the iris. The modification to which I refer, and which, I believe, was first introduced by GRÆFE and MOOREN, consists in tearing out a portion of the iris, as in the operation for artificial pupil. This enlarges the opening through the iris sufficiently to permit the passage of the lens without seriously stretching the circular fibres of the iris. Moreover, the facility for removing all the fragments of the cortical substance is increased. The extraction should be performed in the usual way, at a period of about four weeks after the preliminary operation upon the iris. This modification in extraction of cataract has been highly recommended by several eminent ophthalmic surgeons. I have operated in four instances in the manner described, in all of which, the most favorable results of the operation were, undoubtedly, to a great extent dependent upon the *readiness* with which *every portion of the lens* was removed.

After operations of extraction, I have, for some time, followed GRÆFE'S advice, to cover the eye with a soft cushion of lint, equally distributed over the whole opening of the orbit, and hold it quite firmly in place, by means of a band around the head. This serves to retain the edges of the cornea in place; to prevent general congestion of the eye, and *especially of the choroid*, which is liable, after such operations, to suffer congestion and inflammation, in consequence of the reduction of intra-ocular pressure.

There are two or three subjects of general interest to the profession, relative to the cultivation of ophthalmic science, to which I would call your especial attention:—

Since we last met, there has been organized, in New York, an Ophthalmological Society, composed of many well-known oculists of our country. There is talent and experience among its members, sufficient to secure the annual publication of most valuable monographs, which cannot but awaken increased general interest in our profession in ophthalmic science.

During the past year, the general government has established and supported in Chicago, a large hospital for the treatment of soldiers affected with diseases of the eye and ear. This hospital secures for medical students most valuable opportunities for receiving clinical instruction, at the lectures of the surgeon in charge, Dr. J. S. HILDRETH.

The Chicago Charitable Eye and Ear Infirmary, No. 16 East Pearson St., continues with increased prosperity. The institution has come in possession of a commodious hospital building. Liberal contributions, principally from benevolent citizens of Chicago, have been made for its support. Since the opening of the Dispensary of the Infirmary, in 1858, more than two thousand two hundred poor patients have availed themselves of the treatment provided by the institution. The Infirmary has been incorporated by a special act of the General Assembly of the State of Illinois. The facilities for clinical instruction will be extended, as the growth of the institution may permit.

